# Publisher Correction: Spatial planning with long visual range benefits escape from visual predators in complex naturalistic environments

**DOI:** 10.1038/s41467-020-17398-9

**Published:** 2020-07-14

**Authors:** Ugurcan Mugan, Malcolm A. MacIver

**Affiliations:** 1 Center for Robotics and Biosystems, Northwestern University, Evanston, IL USA; 2 Department of Biomedical Engineering, Northwestern University, Evanston, IL USA; 3 Department of Mechanical Engineering, Northwestern University, Evanston, IL USA; 4 Department of Neurobiology, Northwestern University, Evanston, IL USA

**Keywords:** Computational neuroscience, Ecology, Evolution, Decision, Learning algorithms

Correction to: *Nature Communications* 10.1038/s41467-020-16102-1, published online 16 June 2020.

The original version of this Article contained an error in Ref. 4, which omitted the title of the paper in the edited volume: Yamakita, T. & Miyashita, T. in *Integrative Observations and Assessments, Ecological Research Monographs*, 131–148 (eds Nakano, S.-i., Yahara, T. & Nakashizuka, T.) (Springer Japan, Tokyo, 2014). The correct form of Ref. 4 is: Yamakita, T. & Miyashita, T. Landscape mosaicness in the ocean: its significance for biodiversity patterns in benthic organisms and fish. in *Integrative Observations and Assessments, Ecological Research Monographs*, 131–148 (eds Nakano, S.-i., Yahara, T. & Nakashizuka, T.) (Springer Japan, Tokyo, 2014). This has been corrected in the PDF and HTML versions of the Article.

