# Supplementary material for: Spatial planning with long visual range benefits escape from visual predators in complex naturalistic environments
Source: Nat Commun. 2020 Jun 16;11:3057. doi: 10.1038/s41467-020-16102-1 (PMC7298009; doi:10.1038/s41467-020-16102-1)
Supplement: Supplementary file 3 — Reporting Summary [file 41467_2020_16102_MOESM3_ESM.pdf]

nature research

Corresponding author(s): Malcolm A. MacIver

Last updated by author(s): Apr 2, 2020

Reporting Summary

Nature Research wishes to improve the reproducibility of the work that we publish. This form provides structure for consistency and transparency in reporting. For further information on Nature Research policies, see Authors & Referees and the Editorial Policy Checklist .

Statistics

For all statistical analyses, confirm that the following items are present in the figure legend, table legend, main text, or Methods section.

n/a	Confirmed	
☐	☒	The exact sample size (n) for each experimental group/condition, given as a discrete number and unit of measurement
☐	☒	A statement on whether measurements were taken from distinct samples or whether the same sample was measured repeatedly
☐	☒	The statistical test(s) used AND whether they are one- or two-sided<br>*Only common tests should be described solely by name; describe more complex techniques in the Methods section.*
☐	☒	A description of all covariates tested
☐	☒	A description of any assumptions or corrections, such as tests of normality and adjustment for multiple comparisons
☐	☒	A full description of the statistical parameters including central tendency (e.g. means) or other basic estimates (e.g. regression coefficient) AND variation (e.g. standard deviation) or associated estimates of uncertainty (e.g. confidence intervals)
☐	☒	For null hypothesis testing, the test statistic (e.g. F, t, r) with confidence intervals, effect sizes, degrees of freedom and P value noted<br>*Give P values as exact values whenever suitable.*
☒	☐	For Bayesian analysis, information on the choice of priors and Markov chain Monte Carlo settings
☒	☐	For hierarchical and complex designs, identification of the appropriate level for tests and full reporting of outcomes
☐	☒	Estimates of effect sizes (e.g. Cohen's d, Pearson's r), indicating how they were calculated

Our web collection on statistics for biologists contains articles on many of the points above.

Software and code

Policy information about availability of computer code

Data collection	Data collection was done through simulations written in Python (2.7). Monte-Carlo planning for large POMDPs used to simulate planning was based on the proceeding with the same title written by Silver et al., 2010. Their custom code in C++ is available to download at http://www0.cs.ucl.ac.uk/staff/d.silver/web/Applications.html (Real-time planning in games with hidden state, using partially observable Monte-Carlo planning (POMCP), v1.0). For this study we have largely transfered that code over to Python. Code for habit-based action selection and the hybrid system was custom written in Python. Habit-based action selection based on the PRQL algorithm was custom written using the detailed algorithm provided in the paper.<br><br>Prey and predator visibility in Simulation 2: pseudo-terrestrial experiments was based on Bresenham's line algorithm that determines the points that should be selected for to approximate a straight line between two points on an n-dimensional raster. This is a common approach to draw line primitives in for example a bitmap image. Custom code was written to carry out this operation.<br><br>Lacunarity analysis was carried out using ImageJ (v1.52q) and the FracLac plugin (v2.5) for both the generated environments and the example binary environments.<br><br>As stated in the "Code Availability" section, the code will be made available at https://github.com/MacIver-Lab/gridworld-decisionmaking/. All parameters are provided in Supplementary Tables 2, 3 & 4, along with notes on what we expect the variation will be due to change in the listed parameters.
Data analysis	Data analysis was done in Python 3.7.4, R 3.6.1, and Mathematica 11.1. Network analysis for visually-occlusive spatial complexity and eigencentrality were done in R and Mathematica with 'igraph' package (R v1.2.4.2, Mathematica v0.3.116), and in Python with 'networkx' (v2.3). Statistical analysis was done in Python using 'numpy' (v1.17.2) and 'scipy' (v1.3.1). Videos from raw episode files were created in Matlab.

For manuscripts utilizing custom algorithms or software that are central to the research but not yet described in published literature, software must be made available to editors/reviewers. We strongly encourage code deposition in a community repository (e.g. GitHub). See the Nature Research guidelines for submitting code & software for further information.

Data

Policy information about availability of data

All manuscripts must include a data availability statement. This statement should provide the following information, where applicable:

- Accession codes, unique identifiers, or web links for publicly available datasets
- A list of figures that have associated raw data
- A description of any restrictions on data availability

The source data underlying Figs 2, 3, 4, and 5; Supplementary Figures 3, 4, 5, 6, 7, 8, 9, 10, 11 and Supplementary Table 1 are provided as a Source Data file. Raw episode files used to generate the videos are provided within the Source Data folder. Data to generate all the figures will be available at https://github.com/MacIver-Lab/gridworld-decisionmaking/.

Field-specific reporting

Please select the one below that is the best fit for your research. If you are not sure, read the appropriate sections before making your selection.

☐ Life sciences ☐ Behavioural & social sciences ☒ Ecological, evolutionary & environmental sciences

For a reference copy of the document with all sections, see nature.com/documents/nr-reporting-summary-flat.pdf

Ecological, evolutionary & environmental sciences study design

All studies must disclose on these points even when the disclosure is negative.

Study description	This study looks at the interactions between visual range and environmental complexity on benefiting planning during visually guided predator-prey interactions. Our study is comprised of two large scale simulations that aim to elucidate the advantages and success of habit- and plan-based action selection in aquatic and terrestrial environments, respectively. In our pseudo-aquatic simulations (as detailed in the Methods section) we have randomly selected a predator location, set the number of states the prey was able to forward simulate and prey's visual range for plan-based action selection. These simulations were run for 100 episodes to calculate survival rate, a proxy for quantifying advantage of the tested controller. In our pseudo-terrestrial simulations (as detailed in the Methods section) we randomly generated environments with occlusions up until a predetermined level of clutter density, set the number of states the prey was able to forward simulate, and randomly selected a predator location. These simulations were ran for SO episodes to calculate survival rate. For both of these simulations during habit-based action selection, as per previous studies, the prey utilized the successful paths obtained during plan-based action selection phase. Similar to before, in pseudo-aquatic simulations 100 episodes were run, and in pseudo-terrestrial simulations SO episodes were run.
Research sample	Not applicable
Sampling strategy	No sample size calculation was carried out. We chose our sample sizes based on computational limitations.
Data collection	Data was simulated. .
Timing and spatial scale	Not applicable.
Data exclusions	No exclusions.
Reproducibility	Data was simulated.
Randomization	In the pseudo-aquatic simulations the predator location was randomized (n = 20). In the pseudo-terrestrial simulations the placement of occlusions (n = 20) and predator start location (n = 5) were randomized.
Blinding	Not applicable.

Did the study involve field work? ☐ Yes ☒ No

Reporting for specific materials, systems and methods

We require information from authors about some types of materials, experimental systems and methods used in many studies. Here, indicate whether each material, system or method listed is relevant to your study. If you are not sure if a list item applies to your research, read the appropriate section before selecting a response.

Materials & experimental systems

n/a	Involved in the study
x	Antibodies
x	Eukaryotic cell lines
x	Palaeontology
x	Animals and other organisms
x	Human research participants
x	Clinical data

Methods

n/a	Involved in the study
x	ChIP-seq
x	Flow cytometry
x	MRI-based neuroimaging
