# Supplementary material for: Spatial planning with long visual range benefits escape from visual predators in complex naturalistic environments
Source: Nat Commun. 2020 Jun 16;11:3057. doi: 10.1038/s41467-020-16102-1 (PMC7298009; doi:10.1038/s41467-020-16102-1)
Supplement: Supplementary file 4 — Description of Additional Supplementary Files [file 41467_2020_16102_MOESM4_ESM.pdf]

Description of Additional Supplementary Files

File Name: Supplementary Movie 1
Description: **Representative episodes showing prey behavior with varying visual ranges under plan-based action selection.** For each visual range (except visual range 1) a representative survival and death trial are shown. In open environments, the typical form of successful strategies observed independent of visual range is a positive taxis toward boundaries or solid objects (thigmotaxis), in this case wall-following behavior. At high visual ranges, the prey can quickly correct initially incorrect actions it took (going towards the predator). These trials are representative examples, for full survival paths seen with prey with varying visual ranges see Supplementary Figure 3. Representative example of behaviors observed in a single mid-entropy environment (for full survival paths see Supplementary Figure 6 row 2) with varying visual range is shown. Similar to open environments, across all tested visual ranges (1, 3, and 5) successful strategies can be characterized as wall-following/obstacle-following behavior. Source data are provided as episode files.

File Name: Supplementary Movie 2
Description: **Representative episodes showing prey behavior with varying visual ranges and varying environmental complexity under habit-based action selection.** For visual ranges 3 and 5, a representative survival and death trial are shown in open environments when the prey uses habit-based action selection. Despite the prey not being able to react to the current predator location, we see no significant difference in survival rate between prey that use planning and prey that use habit-based control (Fig. 2d). For low, mid, and high entropy, a representative survival and death trial and shown. In mid-entropy environments complex behaviors such as hiding, that are a direct result of the updated predator location, causes habit-based action selection to fail (Fig. 3j). Source data are provided as episode files.

File Name: Supplementary Movie 3
Description: **Representative strategies in simple (low (0.0–0.3) and high (0.7– 0.9) entropy) environments showing stereotypical behavior.** For each entropy a representative survival and death trial are shown. The typical form of successful strategies observed in simple low entropy environments, which can be characterized as wall-following behavior, or thigmotaxis. In high entropy environments, due to the presence of clutter there is a divergence from the wall-following behavior. However, survival strategies remain stereotyped. These trials are representative examples for each environment, for a representation that includes all others see Supplementary Figure 5 (low entropy = 0.0–0.3; high entropy = 0.7–0.9). Source data are provided as episode files.

File Name: Supplementary Movie 4
Description: **Representative mid-entropy strategies that emerge with plan-based action selection.** Two different survival strategies for two different mid entropy environments are shown (environment #1: entropy = 0.4, environment #2: entropy = 0.5). Additionally, a death trial is shown for these environments. In mid-entropy environments we observe a diversity of prey strategies even when the predator start location is kept constant. Notably, these episodes depict flexible behaviors that are generated to strategically use occlusions based on the current predator strategy. Moreover, we show two different strategies that emerge in a single environment (entropy = 0.5) as a result of variable predator start location. These videos show different strategies that the prey takes (roundabout around occlusions, broken-wing, hiding). Source data are provided as episode files.
