# Supplementary figures and images for: Spatial planning with long visual range benefits escape from visual predators in complex naturalistic environments
Source: Nat Commun. 2020 Jun 16;11:3057. doi: 10.1038/s41467-020-16102-1 (PMC7298009; doi:10.1038/s41467-020-16102-1)

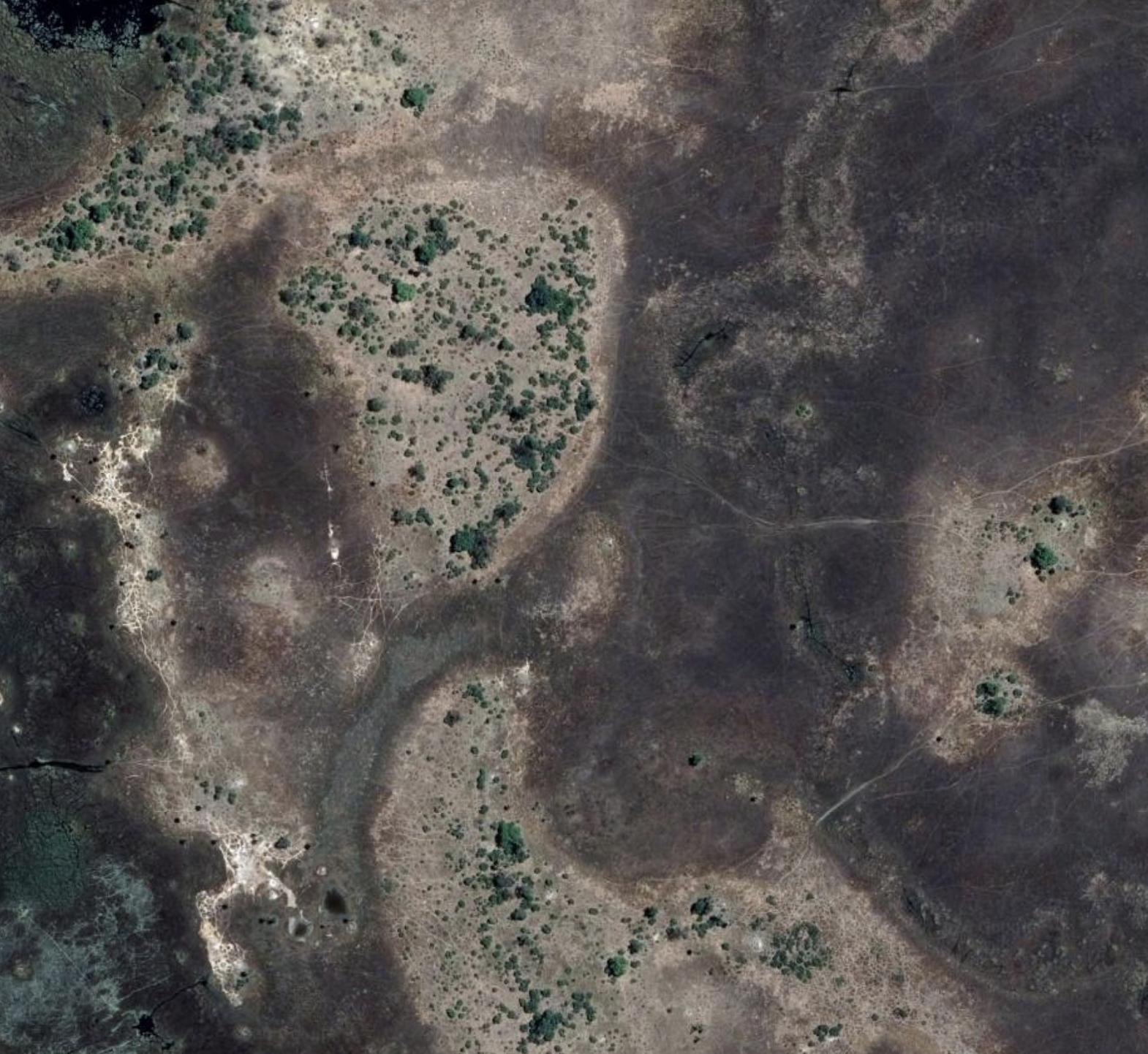

Supplement: Supplementary file 9 — Source Data [file 41467_2020_16102_MOESM9_ESM.zip › Source Data/Okavango Source and Binary Images/GE_Okavango_HILAC.png]

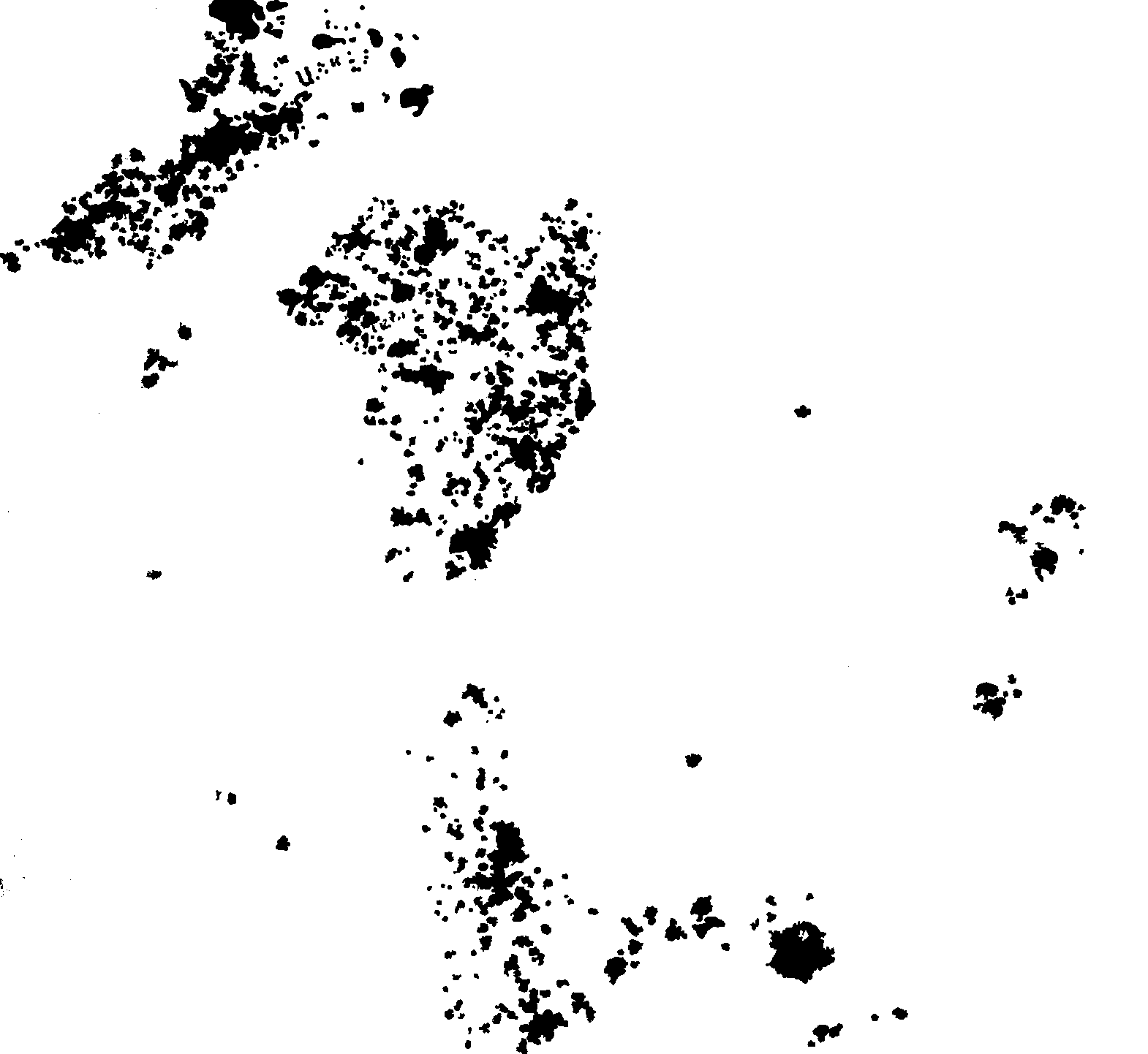

Supplement: Supplementary file 9 — Source Data [file 41467_2020_16102_MOESM9_ESM.zip › Source Data/Okavango Source and Binary Images/Binary_Okavongo_HILAC.png]

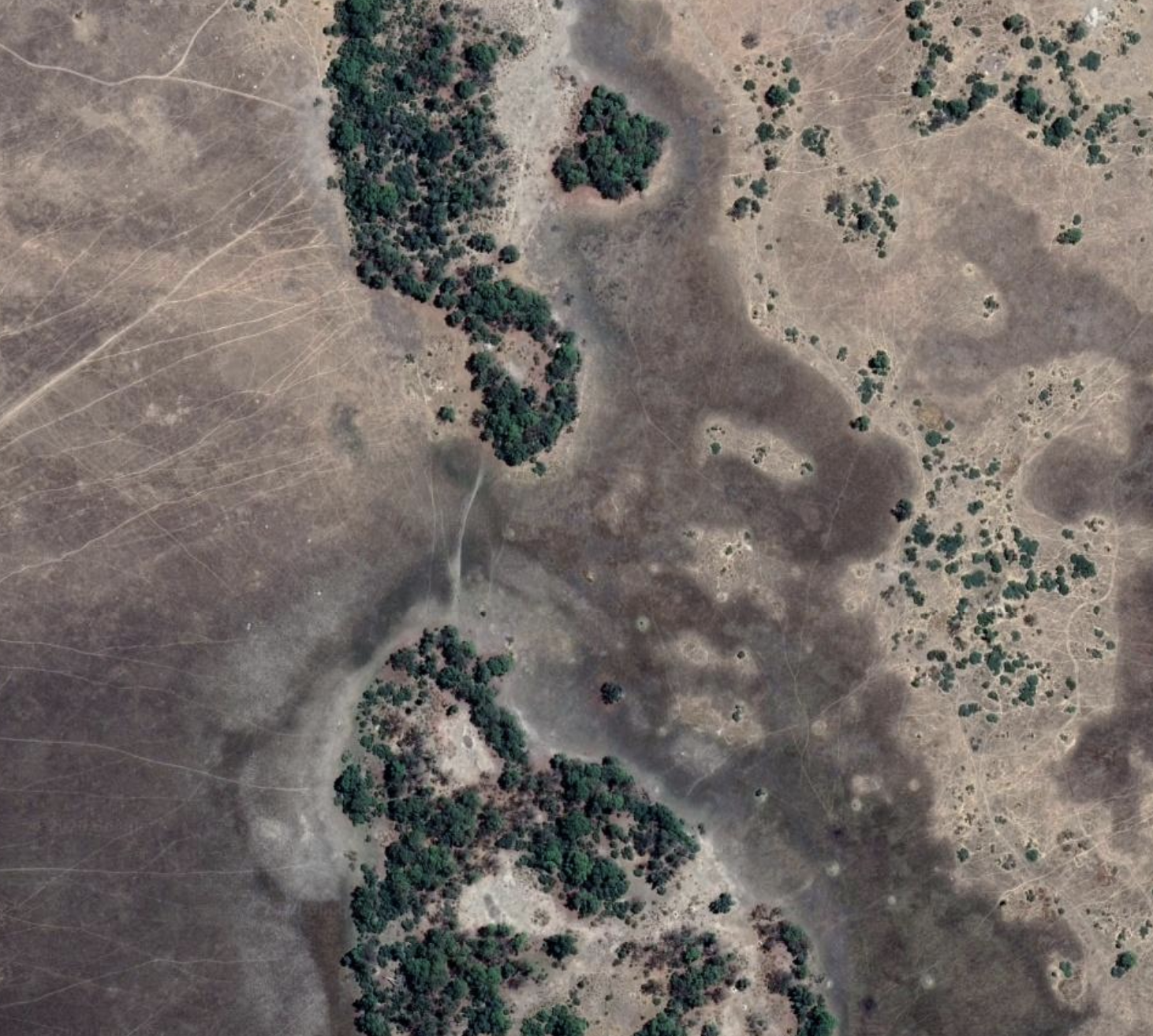

Supplement: Supplementary file 9 — Source Data [file 41467_2020_16102_MOESM9_ESM.zip › Source Data/Okavango Source and Binary Images/GE_Okavango_LOLAC.png]

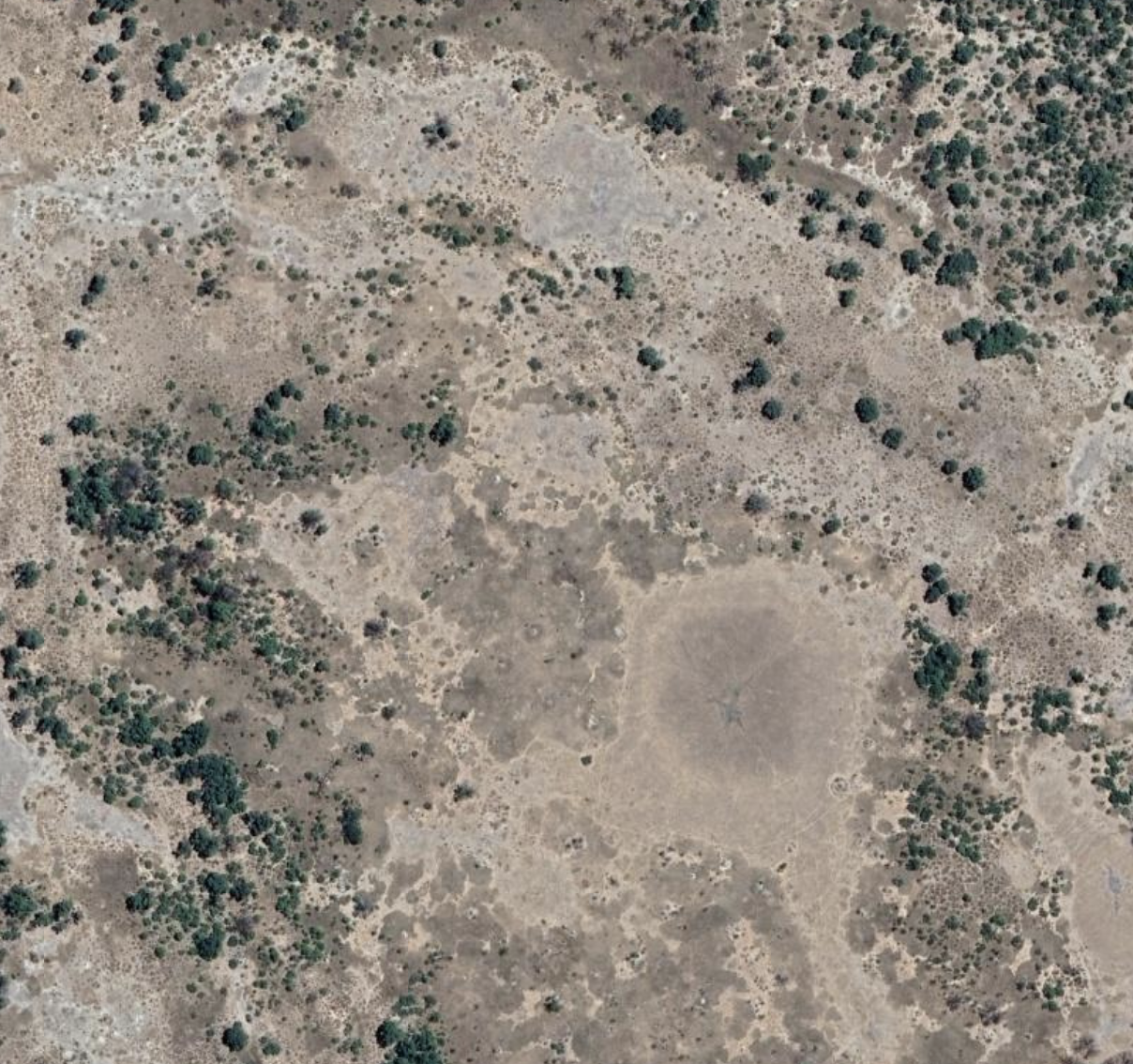

Supplement: Supplementary file 9 — Source Data [file 41467_2020_16102_MOESM9_ESM.zip › Source Data/Okavango Source and Binary Images/GE_Okavango_MIDLAC.png]

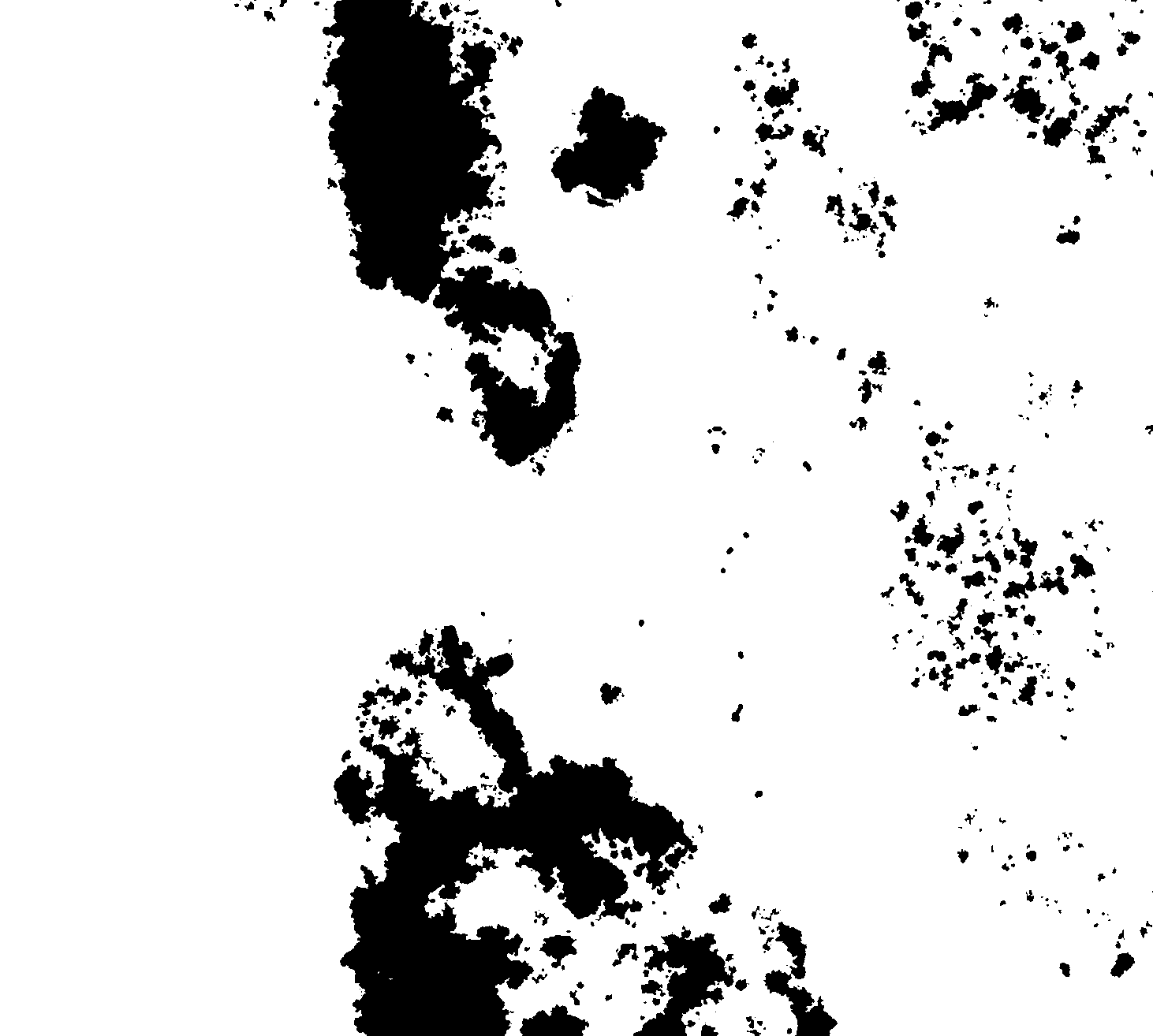

Supplement: Supplementary file 9 — Source Data [file 41467_2020_16102_MOESM9_ESM.zip › Source Data/Okavango Source and Binary Images/Binary_Okavongo_LOLAC.png]

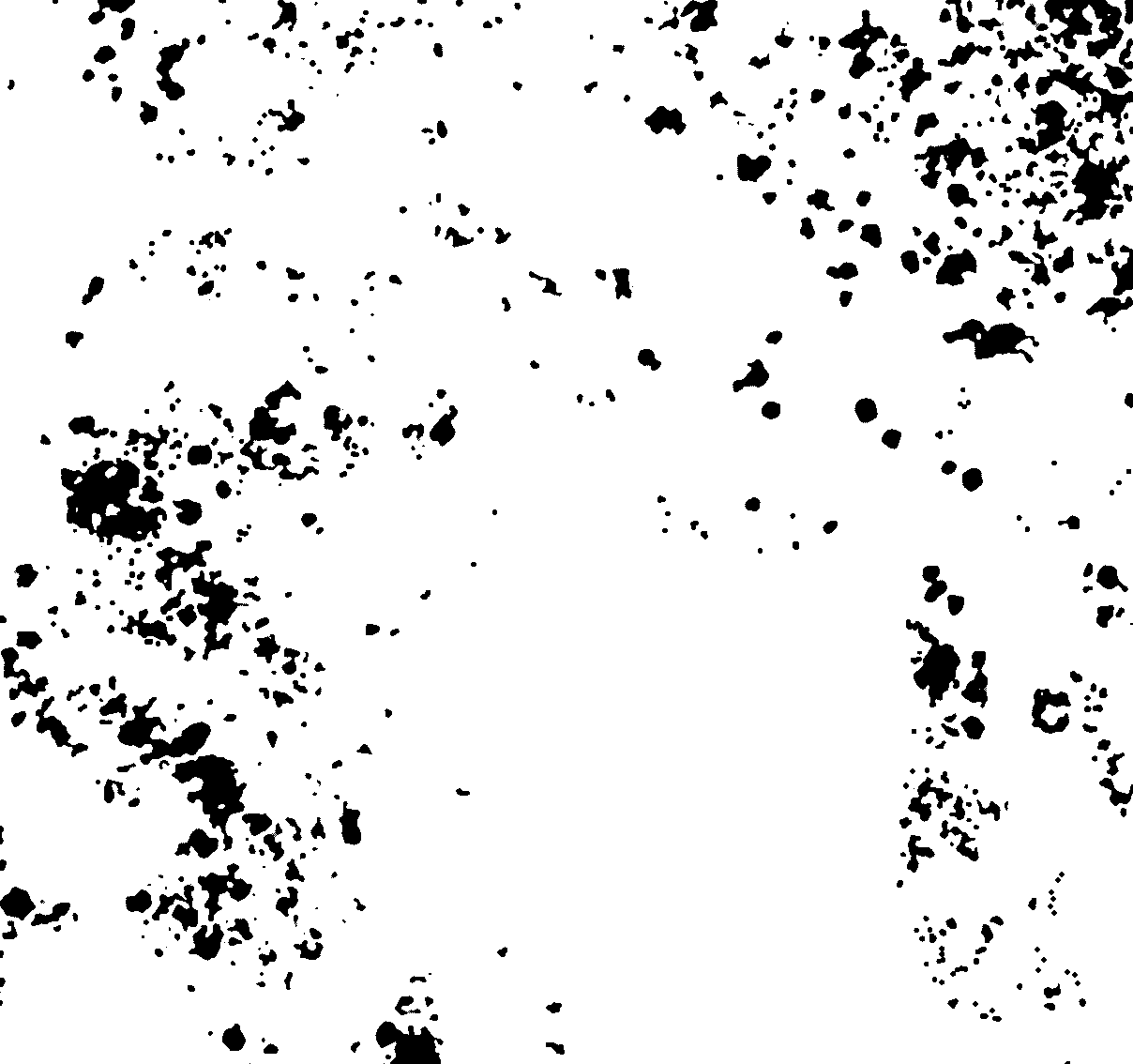

Supplement: Supplementary file 9 — Source Data [file 41467_2020_16102_MOESM9_ESM.zip › Source Data/Okavango Source and Binary Images/Binary_Okavango_MIDLAC.png]
