# Supplementary material for: Spatial planning with long visual range benefits escape from visual predators in complex naturalistic environments
Source: Nat Commun. 2020 Jun 16;11:3057. doi: 10.1038/s41467-020-16102-1 (PMC7298009; doi:10.1038/s41467-020-16102-1)
Supplement: Supplementary file 1 — Supplementary Information [file 41467_2020_16102_MOESM1_ESM.pdf]

Supplementary Information for

Spatial planning with long visual range benefits escape from visual predators in complex naturalistic environments

Mugan & MacIver

Supplementary Methods 1
Supplementary Notes 1–4
Supplementary Discussion 1–3
Supplementary Figures 1–11
Supplementary Tables 1–4
Supplementary References

Supplementary Note

1 Cognitive map assumptions for both the predator and the prey

In the case of our assumption that the predator and prey have a perfect cognitive map (in the predator's case, excluding the location of the safety point that the prey is trying to reach), our modeling choice is driven by a desire to surgically isolate the relative properties of planning versus habit. First, the assumption that the predator has a perfect cognitive map (minus the prey's goal) is the most detrimental to the prey's survival. Here we consider three different cases and their possible outcomes under different levels of cognitive map fidelity.

Case 1: No cognitive map. Neither the predator nor the prey has a cognitive map, and instead, the prey learns a cognitive map by acting and interacting with its environment. Without a model to begin with, the prey agent can not use its internal model to reason about the future and to seek positive outcomes while avoiding the adverse consequences of trial-and-error in the real environment. During the building of the model, we would have to make a large number of empirically poorly-justified modeling choices on learning rates, and the number of exposures to a given random environment to enable the planning algorithm to use a high fidelity model to reason with. The predator in such a case could not be a simple reactive agent, since similar to the prey, the predator would have to learn the environment before choosing its actions. This would cause both the prey and the predator to be learning agents and would necessitate the predator to be more than just a reflex agent.

Case 2: Inaccurate cognitive map updated with learning. Similar to *Case 1*, this scenario would add many more modeling choices. Methods for planning or model-based learning that are robust against model imperfections is an active research area[18]. In such cases, the prey would still use its own model to make decisions but would also have to reason about its imperfect predictions. Given the way in which we have implemented planning, this would cause many actions to be based on a possibly incorrect value function that corresponds to this inaccurate cognitive model. Therefore the decisions would, in the limit of number states forward simulated, not be optimal. If the decisions fail to be optimal, in the context of this scenario (irreversibility since death occurs upon capture), there may not be much to learn; but inasmuch as learning can occur, the issues raised about the complexity of our study and poorly justified parameters enter in. If there is no learning within this case, the decisions of the prey will not reach optimality over time. The predator may not be as affected by the imperfect cognitive map. However, the amount of imperfect information about the location of the occlusions could cause it to get stuck in regions, decreasing its threat to the prey.

Case 3: Perfect cognitive map. The attraction of this choice for our study is avoiding uncertain parameterization of learning the model, and avoiding uncertain parameterization of having an inaccurate model. We believe this enables us to surgically isolate the impact of planning in the context of a generous assumption of learning having previously been completed, and completed with perfect fidelity to the underlying structure (not counting the predator) where that structure is necessarily static (since learning cannot occur to accommodate a dynamic

environment, and a dynamic environment without learning would quickly lead to an inaccurate model—case 2).

2 Visual conditions of the prey and predator

The simulations in which the prey and predator's visual range differ are 1) for the empty world simulations (pseudo-aquatic) (Fig. 1a), and 2) simulations conducted in a set of mid-entropy environments (pseudo-terrestrial) where the prey's visual range was explicitly controlled (1, 3, or 5 cells ahead) (Supplementary Figure 6). In all simulations that included occlusions (pseudo-terrestrial conditions), independent of the prey's visual range (whether full or limited), the prey and predator could not observe one another if there was an occlusion on the line between them. This disconnect between the predator's and the prey's visual range in the aforementioned simulations ensured that there would be observation events between the predator and the prey.

The predator policy in most time steps was aggressive pursuit towards the prey's location. In prior simulations (not shown in the paper), the predator, similar to the prey, had a visual cone in the direction of travel. When the prey was not in view, the predator created a belief distribution of all possible prey locations that were not inside its visual cone (as is done for the prey with respect to the predator). In order to reason about the value of taking an action in these cases, during planning the prey sampled as many predator locations as the number of states forward simulated (100–5000) with probability proportionate to the belief state distribution. For computational expedience, the predator only sampled one prey location proportionate to the belief state distribution, and chose an action to minimize its distance to that sampled prey location. Furthermore, because the predator did not have access to a model for prey action selection (as the prey had for the predator) nor access to the goal location, the pruning and propagation of the predator's belief about the prey location was based on the assumption that the prey acted randomly. This in turn resulted in most episodes to terminate without any observation events (i.e. neither the prey nor the predator ever observed each other) since the predator's actions were effectively random. Given that we did not provide the predator with the location of the goal, or the overall aim of the prey, we could not use a perhaps more natural prey model (e.g. the prey is choosing actions that minimizes its distance to the goal).

Even in the case of a more reasonable prey action selection model for the propagation of the predator's belief distribution (such as using the goal location), we expect that the predator and the prey would only observe each other when the prey was near to the goal position. Therefore, unless the predator was positioned near the prey at the start of the simulation, it would not have enough time steps (actions left) to reach the prey before the prey reached the goal position. The only way to enable the predator to make good decisions, as well as maintain a well supported belief distribution about the prey's location, is to simulate both the prey and the predator as planners. This would allow the predator to choose actions based on more than one sample of the prey location, and model the prey action selection as an independent planner. While this is clearly ideal, it is not computationally tractable given the way planning is implemented (as recognized in the literature[19]): Both the prey and the predator would have to plan an action at each imagined step for each other. However, given the number of action

this idea of hierarchical organization of choices at different levels of temporal abstraction, postulates that planning is used to select subgoals[24]. These selected subgoals act as an internal stimulus that triggers a motor routine or policy. On the other hand, another possibility is that the overall goal acts as a stimulus that triggers a cognitive response of selecting subgoals under the habit system[25]. The actions or policies to reach these subgoals are then chosen through a planning algorithm. There has been a significant amount research on abstract state-space representations that would reduce the size of the original problem (e.g.[26]). Nevertheless, even with such shortcuts, planning is recognized to be demanding and easily degrades into habit-based action selection under time pressure[27].

While the way in which we have implemented plan-based action selection is likely not biologically feasible, it has numerous advantages (e.g. limited number of parameter initializations, theoretical convergence to optimal value function). With such advantages, we expect the approach to provide an upper bound on biological performance that is approximated by more efficient subgoal methods closer to biological feasibility, without altering our key conclusions with respect to visual range and environmental complexity. Finally, tree search model-based methods for Markov decision processes are widely used to model planning in animals[28–32].

Another approach could be to implement simpler planning methods on the value function. It is possible that in environments where we do not see behavioral variability (low and high entropy environments), despite the task featuring a dynamic threat that would conventionally necessitate the relearning of the value function at each time step (essentially, to plan), there is in practice very little change in the value function. Therefore in some sense, while our task is dynamic, it may not be fully dynamic in all environments (which is further evidenced by the success of blind habit in low and high entropy environments). In such rather stable environments, it may be possible to perform entropy minimization over the likelihood of survival, as with infotaxis[33], and simplify the planning process.

Infotaxis chooses actions by minimizing the entropy of a belief distribution representing, say, target location. The planning algorithm presented here optimizes a policy using Bellman backups of a terminal reward. A theorem[34] shows that with sufficient samples, the method converges to the optimal policy. As a result, although these algorithms are distinct, their results could potentially agree if the chosen reward were entropy of a belief. To use infotaxis-like methods here, one would need to use entropy minimization on the belief distribution representing likelihood of survival. However, the complexity of infotaxis (the need to grid the space and compute entropy for all possible actions) makes it impractical to use directly on survival beliefs. Alternatively, bio-inspired algorithms[35–37] that approximate infotaxis in a physiologically plausible manner might be sufficient to show that the rough framework provided by infotaxis can be used for the problems pursued here. More broadly, both infotaxis and the algorithms used here are examples of the more general problem of optimal planning under uncertainty, so one should expect that there will ultimately be many algorithms that lead to similar near-optimal solutions.

These methods can be used in low or high entropy environments where we expect a stable distribution of survival probabilities. However, in mid-entropy environments that feature adjacent

clusters of high and low eigenvector centralities, a possible reason for why we see behavioral variability and the need to plan in regions of high eigenvector centrality is that there is a significant amount of change in the value function after each step of the interaction—analogous to sensorimotor latency or planning cycle duration in animals that plan. This makes the task fully dynamic, and necessitates the re-learning of the value function via planning at each step. Unless short update horizons are used, and robustness to multi-peaked distributions is available (as in ergodic information harvesting[38,39]), infotaxis-like methods may become infeasible.

dissipated 3,000 times faster within water than in air. In addition to this barrier to endothermy underwater, any increase in aerobic capacity of animals that gill rather than breathe has to contend with water having only 1/30th the oxygen of air while being 800 times denser. Gill ventilation therefore requires 800 x 30 = 24,000 times more mass flux assuming identical extraction efficiencies; even with the doubled efficiency of gills, the mass flux is four orders of magnitude higher for respiration with water compared to air[50–52].

Given the disadvantageous energy load due to rapid cooling and the difficulty of recouping this energetic cost through additional oxygen expenditure, for most underwater animals, the heightened velocity of movement, brain power, and sensory performance of endothermy is out of reach. Yet these factors seem to be important for realizing the selective benefit of planning, which also suggests that even if the spatial complexity of underwater environments were sufficient to favor planning in predator-prey contexts, planning may be difficult to evolve in underwater animals or likely to occur in diminished form relative to birds and mammals. A possible exception, discussed above in the section on invertebrate planning, is cephalopods, which may have unusual adaptations for high metabolic rate, including the use of diffusion of oxygen to supply the highly energetic skin display and reception system in addition to gill ventilation. There is evidence, within species of squid, of temperature-corrected rates higher than mammals of similar size[53].

2 The sensory ecology of planning

The specific regime of our study is non-habitizable planning—environments so dynamic that plan-based action selection, with its unique ability to rapidly propagate the value of future actions back to inform what the next action should be, is favored over habit-based action selection. In our study, the key factor advantaging plan-based action selection is the presence of a mid-range level of occlusions. Such occlusions, such as terrestrial features like hillocks[54] and biogenic structure such as shrubs, trees, and grasses, do not typically actively emit signals that can be strategically exploited, but rather are signal reflectors. This makes their use contingent on one of two possibilities: 1) the perceiver has an imaging modality—vision or echolocation; 2) the agent has an accurate and detailed cognitive map in combination with cues from a non-imaging modality (or several in combination) that provides sufficiently precise localization of a moving threat or opportunity. Regarding the first possibility, vision is clearly used by many animals for this purpose (see Discussion). Echolocation, a more recent innovation in mammals[55], provides good resolution and fast update rates but is range-limited by power requirements[56]. Nonetheless, sound propagation through water is as favorable as light propagation through air, so underwater echolocators can gain substantial range advantage over aquatic vision[56]. We would expect our results to generalize to terrestrial mammals engaged in echolocation-guided behaviors provided range is sufficient, and the environment has the appropriate level of lacunarity. It does not appear that aquatic environments meet this criterion. Regarding the second possibility—the use of a cognitive map in combination with non-imaging modalities—our study provisioned an accurate cognitive map to both predator and prey (Methods), so vision was only needed for predator localization. However, predators emit olfactory,

mechanical (substrate vibration), and auditory cues. Given the olfactory dominance of many mammals, including rodents and elephants[57,58], with evidence of cognitive maps[59,60], the use of a cognitive map in combination with a non-imaging modality seems quite likely to occur for survival in non-habitizable contexts. In the absence of a cognitive map, or given an incomplete or inaccurate cognitive map, an animal may need to rely on habit-based action selection or on imaging modalities for planning.

In habitizable planning, where planning generates action sequences initially used for advantage over relatively stable features prior to being shifted over to the habit system, olfaction, audition, path integration, and somatosense are all relevant. Hearing provides range and fast updates, but spatial resolution of sound emitters is generally poor outside of owls that hunt by precise sound localization. Olfaction provides long range, a slow update rate, and variable resolution depending on whether the odor is on a substrate or dispersed in air. It may be immensely important for the generation and indexing of a cognitive map, particularly in many olfaction-dominant mammals such as rodents and elephants[57–61]. Cognitive maps have many uses that are independent of planning, such as present location on a map[62] and corrections to a dead reckoning system[63]. This may result in broader phylogenetic distribution of this trait than planning, with evidence for its presence in fish[41,64,65], in turtles[42], and in insects[66].

3 Planning in invertebrates

The selection and maintenance of behavior is a need of all mobile animals. In terms of habits, the basal ganglia is a crucial structure in vertebrates, and genetic and molecular evidence points to the occurrence of a preceding structure in the last common ancestor of arthropods and vertebrates[67]. Our study suggests that with respect to a visually guided behavior, predator-prey encounters, certain terrestrial environments—those gappy enough to reveal and hide adversaries at long distances (necessitating vision) so that strategizing pays off—confer a selective benefit to planning. A major obstacle for the same set of factors to select for planning in similar conditions for invertebrates is their very low visual acuity, with a median of 0.25 cycles per degree (cpd) for insects and only up to 0.4 cpd for crustaceans[68]. Here, a fascinating exception is provided by two invertebrates, one terrestrial and one aquatic: the jumping spider, at 13 cpd (similar to passerine birds), and cephalopods, at 46 cpd—only lower than primates and raptors. The jumping spider shows evidence of planning while stalking its typically stationary prey, orb-weaver spiders[69,70]. Cephalopods have exceptionally large brains and unusually high metabolic rates (partly accomplished by combining diffusion of oxygen through the skin in addition to gills; one species of squid measured at a temperature-corrected metabolic rate higher than mammals of the same size[53]). The planning abilities of cephalopods have yet to be measured, although it is certainly clear that they have unusually complex cognition[71]. Some have pointed to the complexity of controlling what is, essentially, an infinite-degree of freedom body, a muscular hydrostat with eight arms. A more interesting possibility, relating back to their exceptional visual acuity—which, given the evidence shown in Supplementary Figure 1, cannot be for long range vision—is provided by the fact that their skin is akin to a very high resolution video display, containing millions of neurally controlled pigment cells[72] as well as

phototransduction elements[73,74]. This allows projecting detailed and rapidly changing patterns for camouflage around predators and for deception during sexual competitions[71,75]. During sexual competitions, a male will get between a female and rival, simultaneously displaying the female pattern to the rival on one side of its body and the male pattern to the female on the other side of its body[75]. This is a possible context for a selective benefit of non-habitizable planning without extended sensory range, as it occurs at close quarters with slow changes in position.

In both the camouflage and sexual competition contexts, we can observe a logic similar to that underlying the selective benefit of long-range vision interacting with patchy structure on land: “I see the predator, and if I move there, it will also see me unless I rapidly alter my skin pattern” in the case of camouflage; and “the male thinks I’m a female, and if I move there, it will see that I’m a male unless I rapidly alter my skin pattern” in the case of sexual competitions. The possibility of task logic driving convergence of neural function has been more thoroughly discussed elsewhere[76].
